# N-Terminal Acetylation and C-Terminal Amidation of *Spirulina platensis*-Derived Hexapeptide: Anti-Photoaging Activity and Proteomic Analysis

**DOI:** 10.3390/md17090520

**Published:** 2019-09-04

**Authors:** Qiaohui Zeng, Jianguo Jiang, Jingjing Wang, Qiuchan Zhou, Xuewu Zhang

**Affiliations:** 1 College of Food Science and Engineering, South China University of Technology, Guangzhou 510640, China (Q.Z.) (J.J.); 2 Department of Food Science, Foshan University, Foshan 528000, China; 3 Institute of Laboratory animal science, Jinan University, Guangzhou 510632, China

**Keywords:** photoaging, acetylation, amidation, hexapeptide, proteomics

## Abstract

Ultraviolet (UV) irradiation is a potent inducer for skin photoaging. This paper investigated the anti-photoaging effects of the acetylated and amidated hexapeptide (AAH), originally identified from *Spirulina platensis*, in (Ultraviolet B) UVB-irradiated Human immortalized keratinocytes (Hacats) and mice. The results demonstrated that AAH had much lower toxicity on Hacats than the positive matrixyl (81.52% vs. 5.32%). Moreover, AAH reduced MDA content by 49%; increased SOD, CAT, and GSH-Px activities by 103%, 49%, and 116%, respectively; decreased MMP-1 and MMP-3 expressions by 27% and 29%, respectively, compared to UVB-irradiated mice. Employing isobaric tags for relative and absolute quantitation (iTRAQ)-based proteomics, 60 differential proteins were identified, and major metabolic pathways were determined. Network analysis indicated that these differential proteins were mapped into an interaction network composed of two core sub-networks. Collectively, AAH is protective against UVB-induced skin photoaging and has potential application in skin care cosmetics.

## 1. Introduction

Solar ultraviolet (UV) light is composed of UVC (200–280 nm), UVB (280–315 nm), and UVA (315–400 nm). UVB can penetrate the epidermis and is particularly damaging to skin. It contributes predominantly to skin photoaging. Clinically, photoaging is characterized by coarse solar scars, roughness, dryness, wrinkles, laxity, and pigmentation [1]. UVB-induced photoaging not only damages biological macromolecules such as deoxyribonucleic acid (DNA), carbohydrates, lipids and proteins, but also decrease the activities of antioxidant enzymes in the skin such as superoxide dismutase (SOD) and glutathione peroxidase (GSH-Px) [2]. In addition, UVB-induced photoaging causes damage to the extracellular matrix (ECM) integrity in skin tissues by stimulating the production of various matrix metalloproteinases (MMP) such as MMP-1 and MMP-3 [3].

As early as the year of 2006, the micro alga *Spirulina platensis* extracts was revealed to possess anti-photoaging activity [4]. In addition, Neyrinck et al. showed that the oral administration of a *Spirulina* is able to modulate the gut microbiota and to activate the immune system in the gut, which is a mechanism that may be involved in the improvement of the hepatic inflammation in aged mice [5]. Meanwhile, numerous studies showed that *Spirulina* and *Spirulina*-derived peptides possess multiple activities such as prevention of hyperglycemia [6], antioxidant, immunomodulatory, and anti-inflammatory effects after dietary supplementation [7]. Furthermore, Souza and his research group payed much attention to the benefits of the topical application of *Spirulina*. Their results demonstrated that formulations containing *Spirulina* extract (at 0.1% *w*/*w*) were compatible with the skin and exerted immediate effects on the skin microrelief and hydration. Moreover, their research indicated that *Spirulina* nanoparticles loaded by dimethylmethoxy chromanol could significantly improve the skin pigmentation, the collagen degradation on the dermis and thereby the skin net elasticity, when they were used as supplementation to sunscreen formulation [8]. However, people have paid little attention to the anti-photoaging activity of *Spirulina*-derived peptides.

Recently, we identified a hexapeptide GMCCSR from trypsin hydrolysate of *Spirulina platensis* protein [9]. Preliminary in vitro studies showed that GMCCSR exhibited potent antioxidant and collagen-stimulating activities, and this prompts us to further explore its anti-photoaging activity in UVB-induced mice [9]. However, it is known that chemically synthesized peptides carry free amino and carboxy termini and are electrically charged in general. In order to remove this electric charge, peptide ends are often modified by N-terminal acetylation and/or C-terminal amidation. More importantly, such a modified peptide possesses some advantages: (1) mimics natural peptides due to the absence of the uncharged peptide ends, (2) resists synthetase activity by blocking the peptide ends, (3) increases cell permeability and helps binding to cell membrane, (4) enhances stability toward digestions by aminopeptidases, (5) increases bioactivity and enzyme stability by stabilizing the peptide conformation, (6) enhances activity of peptide hormones by amidation of peptides [10,11,12,13]. Hence, the purpose of this study is to investigate the effects of the acetylated and amidated hexapeptide (AAH) on anti-photoaging in UVB-irradiated Human immortalized keratinocytes (Hacats) and mice, and to understand the mechanism of action by isobaric tags for relative and absolute quantitation (iTRAQ)-based proteomics.

## 2. Materials and Methods

### 2.1. Chemicals

Trypsin was from Promega, Gaungzhou, China. PMSF (Phenylmethanesulfonyl fluoride) was from Solarbio, Guangzhou, China. MTT (3-(4,5-dimethylthiazol-2-yl)-2,5-diphenyltetrazolium bromide), thiocarbamide, DTT (dithiothreitol) and iodoacetamide were obtained from Sigma-Aldrich Co. LLC., Shanghai, China. MDA, GSH-Px, CAT and SOD assay kits were purchased from Nanjing Jiancheng Bioengineering Institute, Nanjing, China. Dulbecco’s modified Eagle’s medium (DMEM), Fetal bovine serum (FBS), and the antibiotic mixture (penicillin-streptomycin) were purchased from Jianyang Biotechnology Co., Ltd., Guangzhou, China. Rabbit anti-Haptoglobin, anti-Cytochrome c, anti-Nucleophosmin, anti-HSP60, anti-CA3, anti-PDHA1, anti-GAPDH were purchased from Bioworlde, Guangzhou, China. Goat anti-rabbit IgG-HRP was from Santa Cruz, CA, USA. Other reagents were of analytical grade.

### 2.2. The Acetylated and Amidated Modification of Hexapeptide

The isolation and purification of *Spirulina platensis*-derived hexapeptide GMCCSR were well described in previous study [9]. GMCCSR and its modification were custom synthesized by Synpeptide Co. Ltd. (Shanghai, China) using the standard Fmoc method by the use of Rink Amide. The purity of synthesized peptides was over 95%.

### 2.3. Cell Viability Using the MTT Method

Human immortalized keratinocytes (Hacats) were purchased from the cell bank of CoBioer biosciences CO., LTD (Nanjing city, China). The cells were cultured in complete DMEM supplemented with 10% fetal bovine serum, 100 IU/mL penicillin, and 100 μg/mL streptomycin, and were incubated at 37 °C in a damp incubator with 5% CO_2_.

Subsequently, the exponentially growing cells (100 µL, 5 × 10^4^ cells/mL) were seeded in 96-well plates for 24 h. Removing the supernatant, cells were washed with phosphate-buffered saline (PBS) and placed in fresh PBS. UVB irradiation of cells was carried out using UV crosslinker (UVP laboratory, Westbury, NY, USA), five parallel tubes emitting 280–320 nm wavelength with a peak at 302 nm, and cells were irradiated at the desired dosages of 35 mJ m^−2^, which was according our earlier publication with minor modification [9]. After UVB treatment, Hacats were incubated in complete DMEM with or without sample treatments. Following 3 days of incubation, medium was removed and 3-(4,5-Dimethylthiazol-2-yl)-2,5-diphenyltetrazolium bromide (MTT) solution (Sigma Aldrich, Los Angeles, CA, USA) was added to each well, and cells were incubated for 4 h at 37 °C. Negative control was MTT solution and culture media, and positive control was Matrixyl, i.e., Palmitoyl Pentapeptide-3: Palmitoyl-KTTKS. Then, 150 μL dimethyl sulfoxide (DMSO) was added to each well and incubated for an additional 10 min. The optical density was read at 490 nm on a SunriseTM microplate reader (TECAN, San Jose, CA, Switzerland).

### 2.4. Animal Experiment

#### 2.4.1. Animal

Female Kunming (KM) mice (7–8 week) were obtained from the Animal Center of Southern Medical University (Guangzhou, China). All experimental procedures were carried out in accordance with the standard guidelines for the care of animals that were approved by Jinan University Committee for Animal Care and Use. Mice were housed at a temperature of 23 ± 2 °C and humidity of 55% ± 10% specific pathogen-free environment with a 12 h light/dark cycle and given free access to standard laboratory diet and water. Prior to the start of the experiment, mice were acclimatized for at least 7 days.

#### 2.4.2. UV Irradiation and Peptide Treatment

UVB has been thought to be responsible for the damaging effects in the skin. Therefore, we used a UVB lamp apparatus (peak at 302 nm) for construction of a photoaged skin animal model in this study. The dorsal skin of mice was exposed to UVB light for 10 weeks of three times per week at an intensity of 60 mJ/m^2^, which was closed to four minimally erythemogenic doses (MED). During the experiment, once erythema, blisters, and erosion occurred in the dorsal skin of mice, the irradiation was stopped for 2–3 days, which could continue until the symptoms disappeared.

We randomly divided 32 KM mice into four groups of eight mice: no UVB exposure (normal), UVB irradiation (model), UVB + Matrixyl (positive control Matrixyl, i.e., Palmitoyl Pentapeptide-3: Palmitoyl-KTTKS), and UVB + AAH groups (experimental group, acetylated and amidated hexapeptide (AAH)).

Hair in dorsal skin of mice was removed within an area of 2.5 × 3 cm^2^ using a lady shaver, and the mice were acclimatized for two days before the experiment. A total of 100 μL of AAH (10 mg/mL), Matrixyl (10 mg/mL), and vehicle (no UVB exposure group and UVB irradiation group) were applied on the shaved dorsal skin. In this study, we used a solvent mixture of ethanol:water:propylene glycol = 3:3:4 (v:v:v) as a vehicle. This solvent mixture is the proper vehicle to fully solve the peptide. We topically applied all samples (100 μL/each mouse) on the dorsal skin of mice, and then gave enough time to be absorbed into the skin (about 2 h). The skin care product Matrixyl was used as the positive control. The mice were exposed to UVB radiation three times per week at 60 mJ/cm^2^ for 10 weeks. We euthanized the mice in a humane way at the end of the study. Mice were anesthetized with ether and then executed cervical dislocation without pain. Then, skin specimens from the central dorsum of the mice were obtained.

#### 2.4.3. Histological Examination and Moisture Content Test of Skin

The dorsal skin of mice was photographed under anesthesia by diethyl ether inhalation at the end of the study. An area of 1.0 × 1.0 cm^2^ was harvested freshly and fixed in 10% neutral buffered formalin. The degree of skin structure alteration and elastosis were assessed microscopically using Haematoxylin-eosin (H&E) staining. To quantify epidermal thickness following UV exposure, measurement was conducted at 10 randomly selected locations per slide using an optical microscope with 200× magnification. Histological alterations were evaluated and quantified through the image analysis program Image Pro Plus 6.0 (Silver Spring, MD, USA).

Skin (0.2 g) was quickly cut and precisely weighted (w_1_), then it was moved into the oven, and dried at 80 °C to constant weight (w_2_). Therefore, the skin moisture percentage could be determined by the following formula: the skin moisture percentage (%) = (w_1_ − w_2_)/w_1_ × 100.

#### 2.4.4. Determination of SOD, CAT, GSH-Px, and MDA in Skin Tissue

The harvested skin tissue (0.4 g) was homogenized (10,000 rpm, 20 s) in nine volumes of 0.9% saline (4 °C) to obtain the 10% skin tissue homogenate. The total supernatant was used for protein content, SOD, CAT, GSH-Px and MDA determination according to the manufacturer’s protocols.

#### 2.4.5. Determination of MMP-1 and MMP-3 in Skin Tissue

Skin tissue (0.4 g) was homogenized (10,000 rpm, 20 s) in nine volumes of PBS (4 °C) to obtain the 10% skin tissue homogenate. The undissolved pellet was removed by centrifugation at 3000× *g* for 20 min at 4 °C, and the total supernatant was saved for the subsequent assays. Secreted MMP-1 and MMP-3 were estimated using ELISA kits (Thermo scientific, Waltham, MA, USA) and protein content was determined according to the manufacturer’s instructions.

### 2.5. Proteomics

#### 2.5.1. Sample Preparation and Labeling

A total of four samples were collected as the target tissue for iTRAQ analysis at Shanghai Majorbio BioMedical Technology Co., Ltd. The samples were lysed to enrich the phosphorylated proteins with phosphoprotein enrichment kit according to the manufacturer’s instructions. The protein concentration was determined by the Bradford method. Afterwards, 150 μg of protein for each sample was mixed with Dithitol (DTT, final concentration of 10 mM), reacted for 30 min at 56 °C, then, Iodoacetamide (IAA, final concentration of 20 mM) was added keeping in darkness for 30 min. Then, five volumes of cold acetone were precipitated for 2 h at −20 °C and then centrifuged at 12,000 rpm for 20 min at 4 °C. The precipitation was dissolved by 20 µL TEAB buffer with 1 M urea. Next, Trypsin (1/50 protein) was added and reacted for 15 h at 37 °C. Trichloroacetic acid (TFA, final concentration of 0.5%) was added to stop the hydrolysis reaction. The deposit was collected and dried by vacuum freeze dryer. Then, the sample (100 μg) was dissolved in dissolution buffer of the iTRAQ kit and reacted with 40 μL reducing reagent and 70 μL Isopropyl alcohol, and then 40 µL Milli-Q water was added after 2 h incubation. All the labeled samples from different treatment groups were pooled and freeze-dried.

#### 2.5.2. Sample Fractionation and LC–MS/MS Analysis

Dried peptides were resuspended with buffer A: water (formic acid and ammonia water was used to adjust the pH to 10). Buffer B was 100% acetonitrile (ACN). Detection wavelengths were set as 214 and 280 nm, meanwhile, flow rate was 200 µL/min. The 60 min gradient comprised of 0%–5% buffer B for 5 min, 5%–25% buffer B for 35 min, 25%–80% buffer B for 5 min, 80% buffer B for 5 min, 80%–100% buffer B for 1 min, and finally 100% buffer B for 9 min. The combined samples were separated into 20 fractions.

Subsequently, the collected fractions were pooled according to the chromatogram profile based on the peak intensity and the products dried in a vacuum for LC–MS/MS analysis.

#### 2.5.3. Data Analysis

The MS/MS spectra were extracted and searched against the database with Uniform Resource Locator (URL) of http://www.uniprot.org/proteomes/UP000000589 using Mascot 2.3.02 software (Boston, MA, USA). Search parameters were set as follows: (1) type of search: MS/MS Ion search; (2) Enzyme: trypsin; (3) Fragment Mass Tolerance: ±0.05 Da; (4) Mass Value: Monoisotopic; (5) Variable modifications: Gln- > pyro-Glu (N-term Q), Oxidation (M), iTRAQ8plex (Y); (6) Peptide Mass Toletance: 10 ppm; (7) Instrument type: Default; (8) Max Missed Cleavages: 1; (9) Fixed modification: Carbamidomethyl (C), iTRAQ8plex (N-term), iTRAQ8plex; (10) Protein Mass: Unrestricted.

The cellular component, molecular function, and biological process were annotated by GO (Gene Ontology) database (http://www.geneontology.org/). The signaling pathways of proteins were elucidated by searching against the Kyoto Encyclopedia of Genes and Genomes database (http://www.genome.jp/kegg/pathway.html). The protein-protein interaction network was analyzed by Search Tool for the Retrieval of Interacting Genes/Proteins (STRING) software (http://string.embl.de/).

#### 2.5.4. Western Blot

The extraction of total protein was carried out according to the extraction method in the experiment of iTRAQ. The quantification of the protein was carried out in strict accordance with the BCA kits. Subsequently, 30 μg of the sample was taken slowly from the micro-injector and added to the sample wells, meanwhile, 5 μL of protein markers were loaded and followed by electrophoresis, transfer, blocking, primary incubation, secondary incubation, and dyeing process. Among them, slightly different conditions were used in the transfer process, the conditions for most antibodies were 300 mA and 40 min, but 300 mA and 60 min for HSP60, 300 mA and 20 min for Cytochrome c. All antibody concentrations during the incubation were 1:1000 dilution. After the dyeing wass finished, pictures were taken or scanned, recording the analysis results.

### 2.6. Statistical Analysis

All assays were carried out in triplicate, and the experimental results were expressed as means ± standard deviations. Statistical analysis was performed by SPSS 16 (SPSS Inc., Chicago, IL, USA). Data were analyzed using the least significant difference (LSD) method by analysis of variance, and the value differences were considered to be significant when *p* < 0.05. The spectrum data from iTRAQ results were submitted for protein identification, and a database search was carried out using ProteinPilot Software 4.5 (AB SCIEX, Seattle, WA, USA) to perform database searches. The database used was the SwissProt_2013_09 (total sequence 540958). The search parameters used were as follows: Cysteine alkylation with MMTS; Trypsin Digestion; Triple TOF 5600; ID focus with Biological modifications; Search effort with thorough ID. A decoy database search strategy was used to determine the false discovery rate (FDR) for protein identification. The criteria for protein identification was set to FDR < 0.1%.

## 3. Results

### 3.1. The Toxicity on Hacats and the Protective Effect on the Damaged Hacats

By MTT assay, the surviving rates of Hacats treated by *Spirulina platensis*-derived hexapeptide GMCCSR, AAH and matrixyl were 99.18% ± 6.97%, 81.52% ± 6.26%, and 5.32% ± 1.83%, respectively. Then, the Hacats were irradiated by UVB, and the damaged Hacats were treated with the peptides again. The surviving rates corresponding to *Spirulina platensis*-derived hexapeptide GMCCSR, AAH and matrixyl treatment were 112.97% ± 10.73%, 137.14% ± 8.42%, and 121.56% ± 1.42%, respectively, and the UVB control was set as 100%. Comparing with the unmodified hexapeptide, the surviving rates of Hacats treated by AAH reduced from 99.18% ± 6.97% to 81.52% ± 6.26%, which was still above 80%. Thus, the follow-up step was to conduct further experiments to explore its protection effects for the UVB-damaged Hacats. In addition, the modified hexapeptide exhibited significantly lower toxicity (*p* < 0.05) and significantly stronger protective effect (*p* < 0.05) on Hacats than the positive control matrixyl.

### 3.2. Morphology, Thickness, and Moisture Content of the Dorsal Skin on Mice

**Figure 1:**
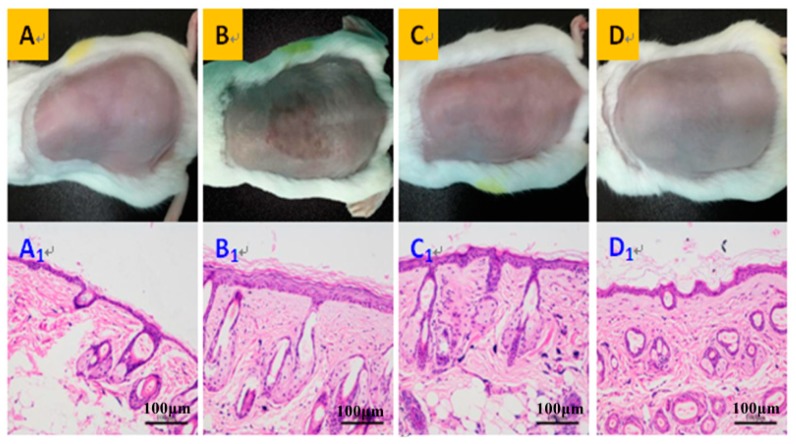
Morphological features (**A**–**D**) and histological examination (**A1**–**D1**, 200×) of the dorsal skin of mice. Normal control (**A,A1**), UVB irradiation group (**B,B1**), positive control matrixyl group (**C,C1**), and the acetylated and amidated hexapeptide group (**D,D1**).

The morphological observation indicated that the dorsal skin of UVB-irradiated mice had distinct features: dark-brown color, deep wrinkle, relaxable surface, and local aging and necrosis appeared on the surface cortex (Figure 1B). After matrixyl treatment, the appearance of skin was improved, but was still a little bit brown, with clear signs of UVB irradiation (Figure 1C). However, the dorsal skin of mice treated with AAH (Figure 1D) was greatly improved, which was better than the positive control matrixyl group, and was most similar to normal group (Figure 1A): smooth surface, rosy color, satiation, no signs of relaxation and wrinkle. Notably, Figure 1(D1) also showed irregular epidermal thickening, and it is possible that there still exists some inflammation. Anyway, the extent of thickening was much smaller than those of the model group (Figure 1(B1)) and positive drug group (Figure 1(C1)), although it was slightly greater than that of the normal group (Figure 1(A1)). Furthermore, the histological examination by haematoxylin-eosin (H&E) staining displayed that the mice skin in the normal group had thin cortex, regularly arranged collagen fibrils, plump subcutaneous hair follicles and sebaceous glands (Figure 1A). The skin in the UVB-irradiation group had irregularly thickened cortex, broken nuclei, vacuolar degeneration of epidermal basal cells, and inflammatory cells (lymphocytes and monocytes) infiltration of the dermis (Figure 1(B1)). In the positive control matrixyl group, although the cortex became thinner, there was still slight vacuolar degeneration and inflammatory cells infiltration (Figure 1(C1)). While in the AAH treatment group, the cortex was further thinned, and followed by disappearance of vacuolar degeneration and inflammatory cells infiltration (Figure 1(D1)). In a word, these results suggest that AAH possessed better effects in improving mice skin than the positive control matrixyl.

**Figure 2:**
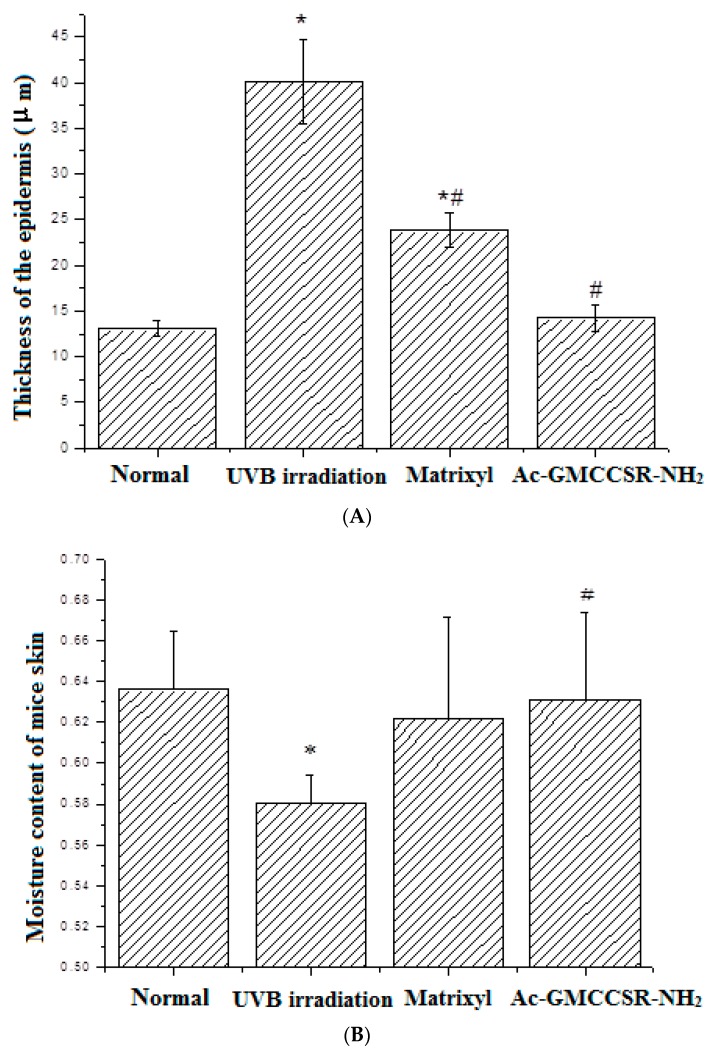
Thickness (**A**) and moisture content (**B**) of the dorsal skin of mice. Normal control, UVB irradiation group, positive control matrixyl group, the acetylated and amidated hexapeptide group (Ac-GMCCSR-NH_2_). * significant compared to normal group, # significant compared to UVB irradiation group.

By measuring the thickness of the dorsal skin (Figure 2), the results showed that UVB irradiation significantly (*p* < 0.05) increased the thickness of skin from 13.1 µm in the normal group to 40.2 µm; the positive control matrixyl decreased 40.2 µm of thickness in the UVB irradiation group to 23.8 µm, but still significantly (*p* < 0.05) greater than 13.1 µm in the normal group; and the thickness of skin in AAH group (13.5 µm) was close to 13.1 µm in the normal group. Further evaluation of moisture content indicated that UVB irradiation significantly (*p* < 0.05) decreased the moisture content (58%), compared to normal group (63.8%); the positive control matrixyl increased the moisture content (62.4%), especially, AAH significantly (*p* < 0.05) elevated the moisture content to 63.2%, compared to UVB irradiation group. This suggests that AAH was also better than the positive control matrixyl in terms of skin thickness and moisture content.

### 3.3. Effects on MDA, SOD, CAT, GSH-Px, MMP-1, and MMP-3 in Skin Tissue of Mice

**Figure 3:**
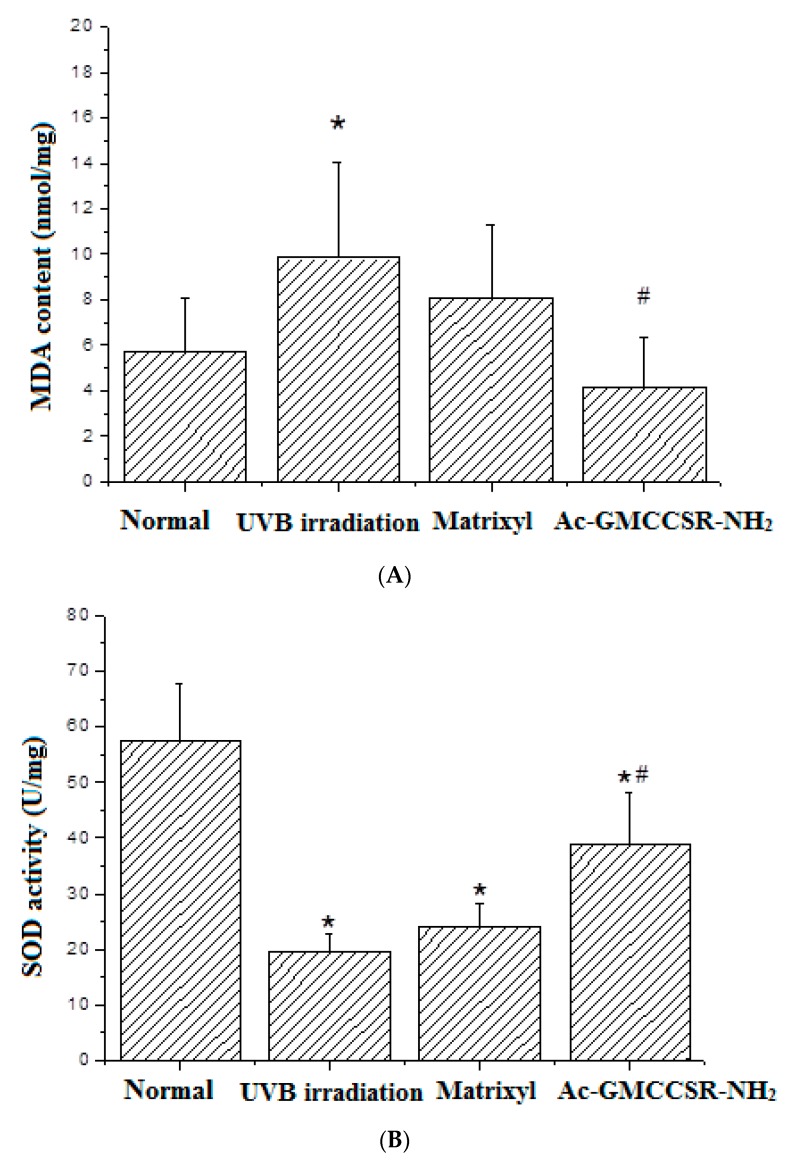

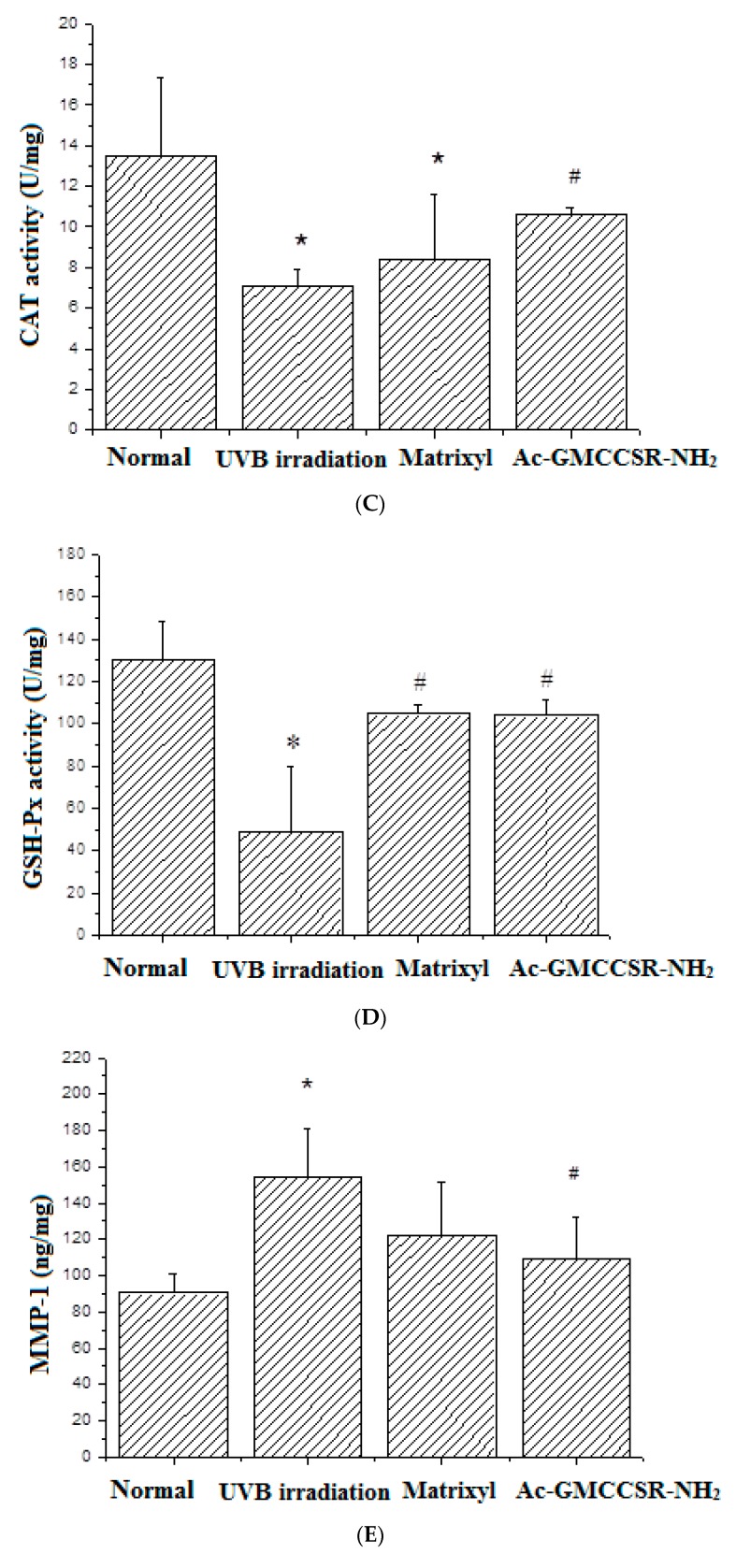

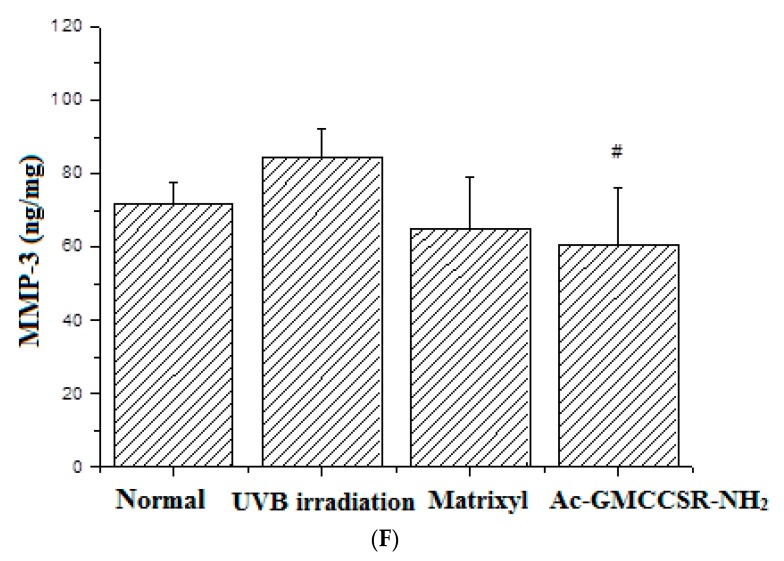
Effects on MDA (**A**), SOD (**B**), CAT (**C**), GSH-Px (**D**), MMP-1 (**E**), and MMP-3 (**F**) in skin tissue of mice. Normal control, UVB irradiation group, positive control matrixyl group, and the acetylated and amidated hexapeptide group (Ac-GMCCSR-NH_2_). * significant compared to normal group, # significant compared to UVB irradiation group.

As shown in Figure 3A, UVB irradiation caused the significant (*p* < 0.05) increase of MDA from 5.8 nmol/mg in the normal mice to 9.8 nmol/mg in UVB-irradiated mice. Matrixyl treatment decreased the increased MDA from 9.8 to 7.9 nmol/mg, while AAH significantly (*p* < 0.05) decreased the increase from 9.8 to 4.0 nmol/mg, up to 49%, even lower than 5.8 nmol/mg in the normal group.

For antioxidant enzymes SOD, CAT, and GSH-Px, there was an identical trend in their alterations: UVB irradiation decreased the activities of SOD, CAT, and GSH-Px, but they were increased in the group of matrixyl treatment and in the group of treatment with AAH. Specifically, the activities of SOD in normal, UVB-irradiation, matrixyl, and AAH treatment groups were 57.5, 19.1, 4.7, and 38.8 U/mg, respectively (Figure 3B); the activities of CAT in normal, UVB-irradiation, matrixyl, and AAH treatment groups were 13.4, 7.1, 8.2, and 10.6 U/mg, respectively (Figure 3C); the activities of GSH-Px in normal, UVB-irradiation, matrixyl, and AAH treatment groups were 130.3, 48.8, 105.1, and 105.2 U/mg, respectively (Figure 3D).

Moreover, Figure 3E shows that the expression of MMP-1 in UVB irradiation group (150.2 ng/mg) was increased compared to normal group (90 ng/mg), but such an increase was slowed down in the matrixyl treatment group (120.1 ng/mg) and the modified hexapeptide treatment group (109.8 ng/mg). Similarly, Figure 3F displayed that the expression of MMP-3 in the UVB irradiation group (82.6 ng/mg) was also increased compared to normal group (71.2 ng/mg), while both matrixyl and the modified hexapeptide decreased the expression of MMP-3 to 63.4 and 58.8 ng/mg, respectively.

### 3.4. Proteomic Analysis of Skin Tissue in Mice

In order to explore molecular mechanism of protective effects on mice skin exerted by AAH, the iTRAQ-based proteomics was performed on the skin tissue from mice treated with UVB irradiation (model group) and with UVB + AAH (experimental group). The results showed that 60 differential proteins were identified, 33 proteins were up-regulated, and 27 proteins were down-regulated proteins (Table 1). Among them, significantly up-regulated proteins include: PYGM_MOUSE (1.9 fold), ODPA_MOUSE (1.6 fold), AT2A1_MOUSE (1.6 fold), TITIN_MOUSE (1.9 fold), ECP3_MOUSE (1.8 fold); significantly down-regulated proteins include: RS21_MOUSE (−1.6 fold), K1C17_MOUSE (−1.8 fold), HPT_MOUSE (−3.4 fold), K1C28_MOUSE (−1.8 fold), K1C27_MOUSE (−1.7 fold), K1C16_MOUSE (−1.6 fold), K2C71_MOUSE (−2.1 fold), K2C6A_MOUSE (−3.2 fold).

**Figure 4:**
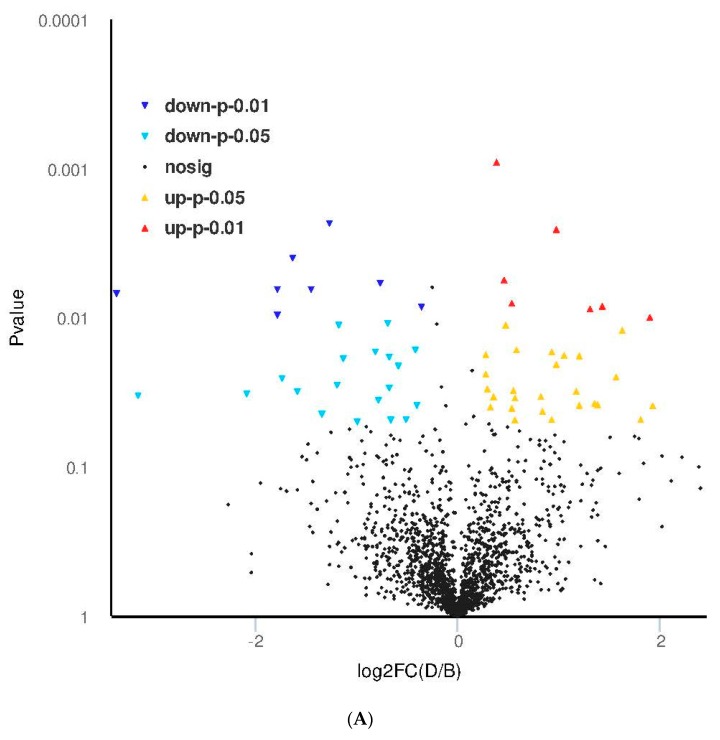

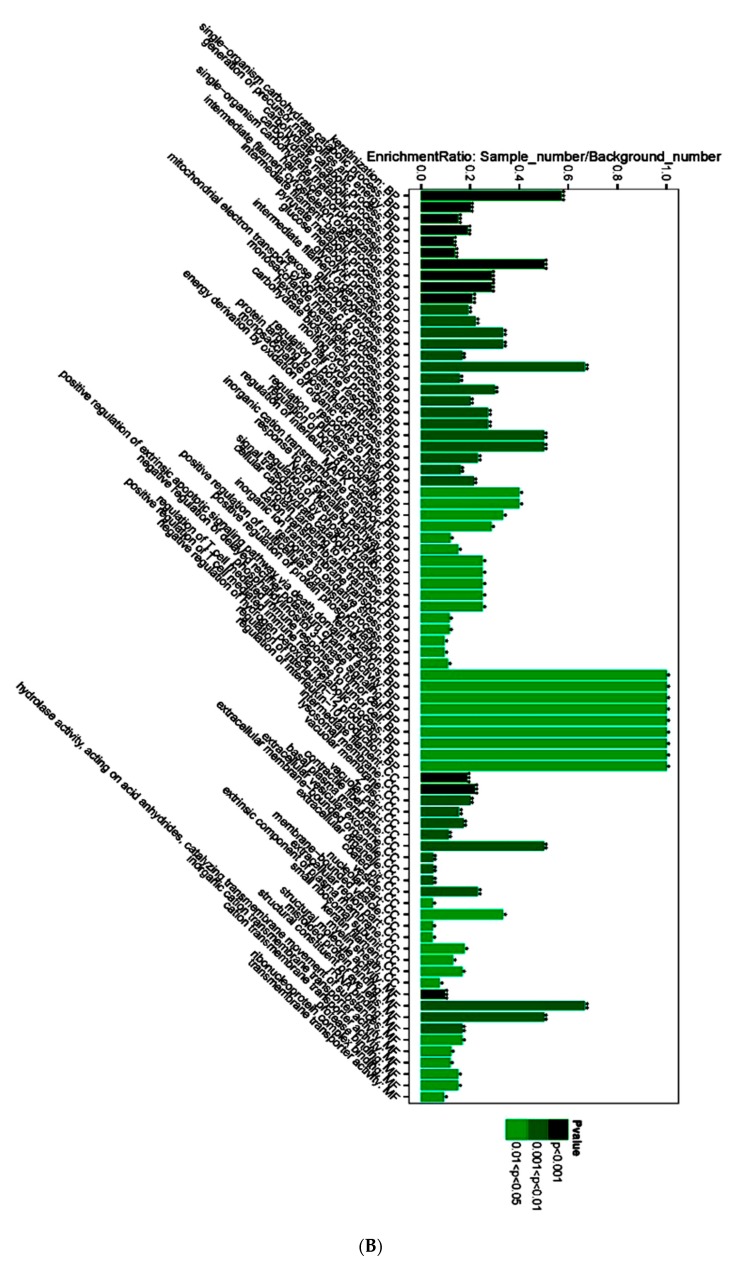

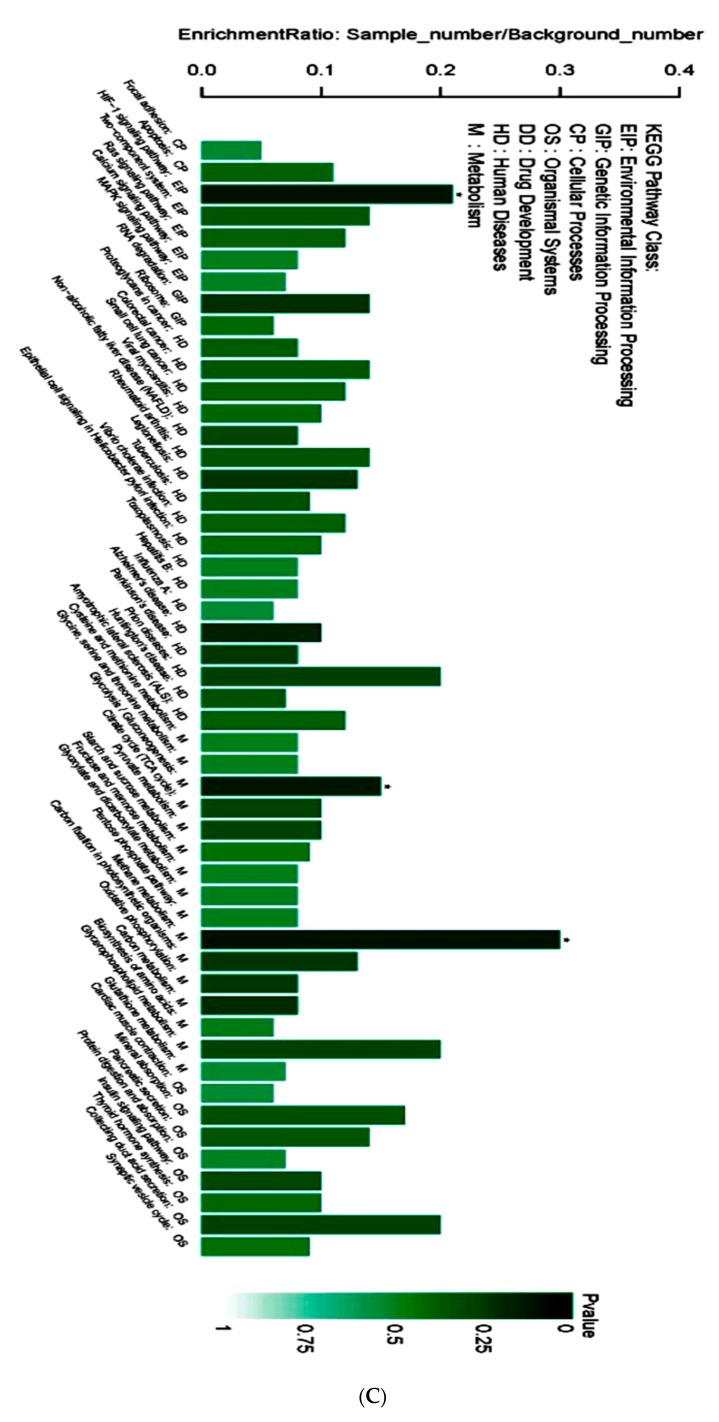

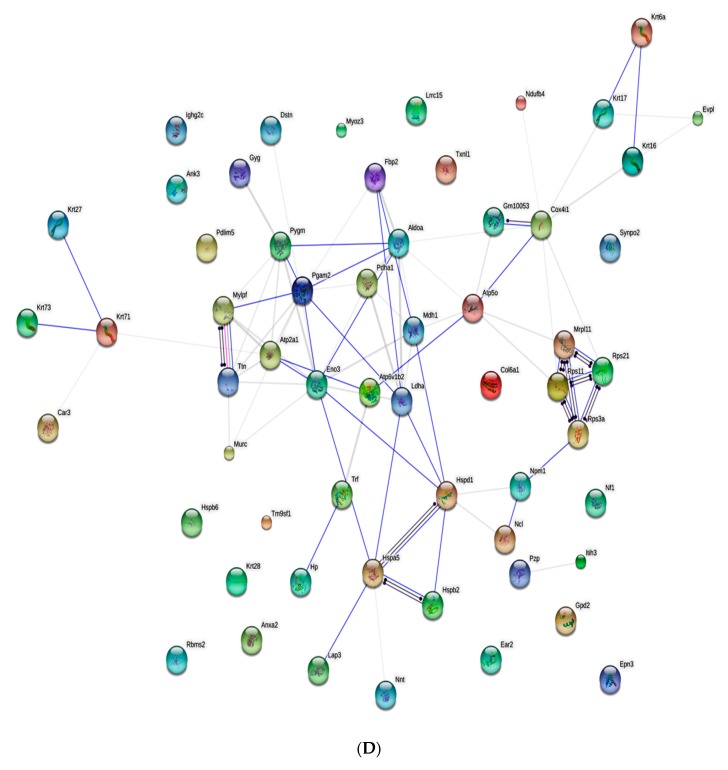

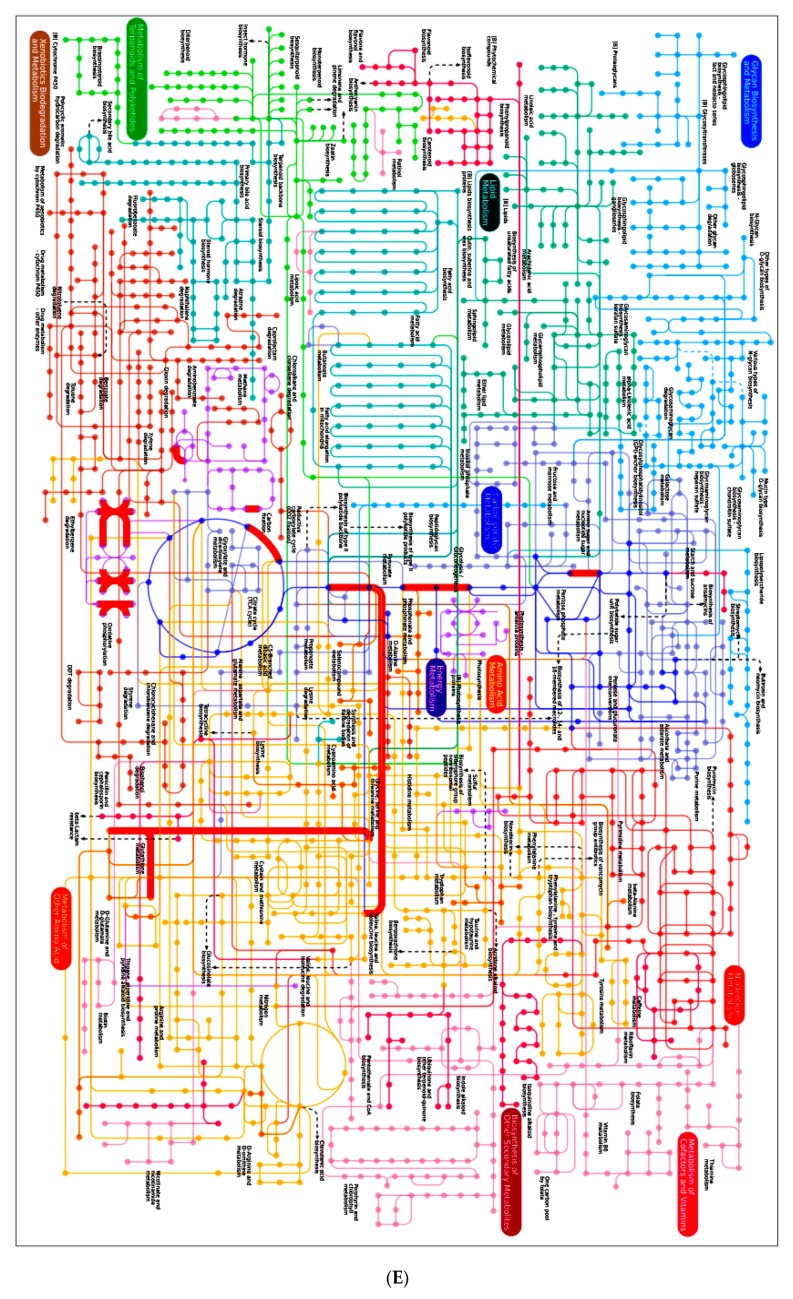
Volcano diagram (**A**), GO enrichment analysis. 1 keratinization: BP; 2 single-organism carbohydrate catabolic process: BP; 3 generation of precursor metabolites and energy: BP; 4 carbohydrate catabolic process: BP; 5 carbohydrate metabolic process: BP; 6 single-organism carbohydrate metabolic process: BP; 7 hair follicle morphogenesis: BP; 8 intermediate filament cytoskeleton organization: BP; 9 intermediate filament-based process: BP; 10 pyruvate metabolic process: BP; 11 glucose metabolic process: BP; 12 glycolytic process: BP; 13 intermediate filament organization: BP; 14 gluconeogenesis: BP; 15 hexose metabolic process: BP; 16 mitochondrial electron transport, cytochrome c to oxygen: BP; 17 monosaccharide metabolic process: BP; 18 hexose biosynthetic process: BP; 19 carbohydrate biosynthetic process: BP; 20 molting cycle process: BP; 21 hair cycle process: BP; 22 regulation of bone resorption: BP; 23 protein targeting to plasma membrane: BP; 24 monosaccharide biosynthetic process: BP; 25 energy derivation by oxidation of organic compounds: BP; 26 response to heat: BP; 27 regulation of nuclease activity: BP; 28 regulation of bone remodeling: BP; 29 regulation of interleukin-6 production: BP; 30 MAPK cascade: BP; 31 inorganic cation transmembrane transport: BP; 32 response to temperature stimulus: BP; 33 Notch signaling pathway: BP; 34 regulation of tissue remodeling: BP; 35 signal transduction by phosphorylation: BP; 36 cellular carbohydrate catabolic process: BP; 37 protein targeting to membrane: BP; 38 cation transmembrane transport: BP; 39 inorganic ion transmembrane transport: BP; 40 response to oxidative stress: BP; 41 positive regulation of multicellular organismal process: BP; 42 positive regulation of protein phosphorylation: BP; 43 fermentation: BP; 44 positive regulation of extrinsic apoptotic signaling pathway via death domain receptors: BP; 45 negative regulation of delayed rectifier potassium channel activity: BP; 46 phosphatidylinositol 3-kinase signaling: BP; 47 regulation of T cell mediated immune response to tumor cell: BP; 48 positive regulation of T cell mediated immune response to tumor cell: BP; 49 negative regulation of hydrogen peroxide metabolic process: BP; 50 regulation of interleukin-12 production: BP; 51 regulation of interleukin-10 production: BP; 52 intermediate filament: CC; 53 lysosomal membrane: CC; 54 vacuolar membrane: CC; 55 Z disc: CC; 56 vacuolar part: CC; 57 contractile fiber part: CC; 58 basal plasma membrane: CC; 59 extracellular vesicular exosome: CC; 60 extracellular membrane-bounded organelle: CC; 61 extracellular organelle: CC; 62 coated pit: CC; 63 vesicle: CC; 64 nucleolar part: CC; 65 membrane-bounded vesicle: CC; 66 extracellular region part: CC; 67 extrinsic component of plasma membrane: CC; 68 small ribosomal subunit: CC; 69 keratin filament: CC; 70 myelin sheath: CC; 71 structural molecule activity: MF; 72 misfolded protein binding: MF; 73 structural constituent of eye lens: MF; 74 rRNA binding: MF; 75 hydrolase activity, acting on acid anhydrides, catalyzing transmembrane movement of substances: MF; 76 inorganic cation transmembrane transporter activity: MF; 77 cation transmembrane transporter activity: MF; 78 protease binding: MF; 79 ribonucleoprotein complex binding: MF; 80 transmembrane transporter activity: MF (**B**). KEGG metabolic pathways. 1 Focal adhesion: CP; 2 Apoptosis: CP; 3 HIF-1 signaling pathway: EIP; 4 Two-component system: EIP; 5 Ras signaling pathway: EIP; 6 Calcium signaling pathway: EIP; 7 MAPK signaling pathway: EIP; 8 RNA degradation: GIP; 9 Ribosome: GIP; 10 Proteoglycans in cancer: HD; 11 Colorectal cancer: HD; 12 Small cell lung cancer: HD; 13 Viral myocarditis: HD; 14 Non-alcoholic fatty liver disease (NAFLD): HD; 15 Rheumatoid arthritis: HD; 16 Legionellosis: HD; 17 Tuberculosis: HD; 18 *Vibrio cholerae* infection: HD; 19 Epithelial cell signaling in *Helicobacter pylori* infection: HD; 20 Toxoplasmosis: HD; 21 Hepatitis B: HD; 22 Influenza A: HD; 23 Alzheimer’s disease: HD; 24 Parkinson’s disease: HD; 25 Prion diseases: HD; 26 Huntington’s disease: HD; 27 Amyotrophic lateral sclerosis (ALS): HD; 28 Cysteine and methionine metabolism: M; 29 Glycine, serine and threonine metabolism: M; 30 Glycolysis/Gluconeogenesis: M; 31 Citrate cycle (TCA cycle): M; 32 Pyruvate metabolism: M; 33 Starch and sucrose metabolism: M; 34 Fructose and mannose metabolism: M; 35 Glyoxylate and dicarboxylate metabolism: M; 36 Pentose phosphate pathway: M; 37 Methane metabolism: M; 38 Carbon fixation in photosynthetic organisms: M; 39 Oxidative phosphorylation: M; 40 Carbon metabolism: M; 41 Biosynthesis of amino acids: M; 42 Glycerophospholipid metabolism: M; 43 Glutathione metabolism: M; 44 Cardiac muscle contraction: OS; 45 Mineral absorption: OS; 46 Pancreatic secretion: OS; 47 Protein digestion and absorption: OS; 48 Insulin signaling pathway: OS; 49 Thyroid hormone synthesis: OS; 50 Collecting duct acid secretion: OS; 51 Synaptic vesicle cycle: OS (**C**). Interaction network identified by STRING (**D**) and by IPATH (**E**) for differential proteins related to the protection effect of the acetylated and amidated hexapeptide.

The volcano diagram of differential proteins is shown in Figure 4A. GO enrichment analysis (Figure 4B) indicated that 13 functions were extremely significantly enriched (*p* < 0.001), for example, keratinization, carbohydrate catabolic and metabolic processes, intermediate filament cytoskeleton organization, hair follicle morphologenesis, lysosomal membrane, etc.; 27 functions were very significantly enriched (*p* < 0.01), for example, glucose metabolic process, hair cycle process, regulation of bone remodeling, mitochondrial electron transport cytochrome c to oxygen, vacuolar membrane, basal plasma membrane, misfolded protein binding, response to heat, etc.; others were significantly enriched (*p* < 0.05), such as positive regulation of apoptotic signaling pathway via death domain receptor, negative regulation of hydrogen peroxide metabolic process, regulation of interleukin-10 and -12 productions, etc.

KEGG (Kyoto Encyclopedia of Genes and Genomes) metabolic pathway analysis (Figure 4C) showed that only three pathways were significantly (*p* < 0.05) influenced: HIF-1 signaling pathway, glycolysis/gluconeogenesis, and methane metabolism. Other important pathways include RNA degradation, Alzheimer’s disease, Leglonellosis, carbon metabolism, mineral absorption, insulin signaling pathway, collecting duct acid secretion, etc. Interaction network analysis by String demonstrated that 42 differential proteins were involved in the acting network of AAH (Figure 4D). Two core sub-networks existed: one was the sub-network centered by Pgam2, including Pygm, Mylpf, Atp2a1, Akdoa, Ldha, etc.; another was the sub-network that consisted of four proteins, i.e., Rps11, Rps3a, Rps21, and Mrpl11. By iPath2.0 (http://pathways.embl.de/ipath2.cgi#) analysis, the global view of the pathways involved in differential proteins is presented in Figure 4E. The major pathways include Oxidative phosphorylation, glutathione metabolism, Cysteine and methionine metabolism, amino sugars and nucleotides metabolism, etc. The differential proteins that mostly participated in these pathways include ODPA_MOUSE (K00161), F16P1_MOUSE (K03841), ENOA_MOUSE (K01689), MDHC_MOUSE (K00025), PGAM1_MOUSE (K01834).

In summary, proteomic analysis revealed that the top regulated proteins include Pygm (1.9 fold), Titin (1.9 fold), Ecp3 (1.8 fold), Hpt (−3.4 fold), K2c7 (−2.1 fold), and K2c6a (−3.2 fold). The significantly enriched functions include (*p* < 0.001) keratinization, intermediate filament cytoskeleton organization, hair follicle morphologenesis and lysosomal membrane; but only three pathways were significantly (*p* < 0.05) regulated: HIF-1 signaling pathway, glycolysis/ gluconeogenesis, and methane metabolism. The network analysis of differential proteins demonstrated the existence of two core sub-networks: one was the sub-network centered by Pgam2; another was the sub-network that consisted of four proteins (Rps11, Rps3a, Rps21, and Mrpl11).

### 3.5. Western Blot Verification

**Figure 5:**
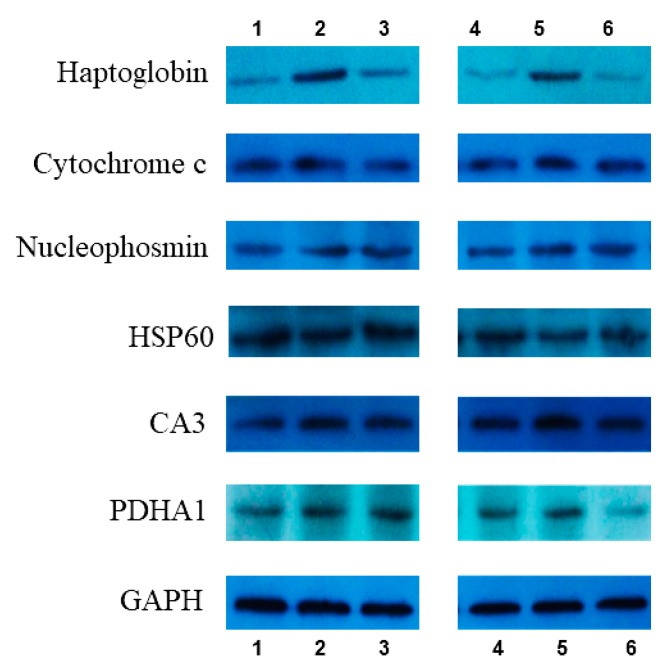
Validation of partly differentially expressed protein by Western blot: 1 and 4 come from the normal group; 2 and 5 come from the model group; 3 and 6 come from the acetylated and amidated hexapeptide group(Ac-GMCCSR-NH2).

As mentioned above, proteomics identified 60 differential proteins between the UVB irradiation group and AAH treatment group. From them, six proteins were selected for verification by Western blot: Haptoglobin, Cytochrome c, Nucleophosmin, HSP60, CA3, PDHA1, and two parallels per sample. Expression intensities were determined by the digital gel image analysis system (LG2000, Hangzhou LongGene Scientific Instrument Co., Ltd. Hangzhou, China). Results revealed that all six proteins displayed consistent alterations between iTRAQ and Western blot experiments (Figure 5 and Table 2).

## 4. Discussion

Except for intrinsic aging, photoaging is a major process of skin aging. Keratinocytes are the outermost layer of the skin, constituting 95% of the cells in the epidermis. UV radiation induces apoptosis of keratinocytes to form sunburn cells, showing premature and abnormal keratinization [14]. Tsoyi et al. [15] proved that anthocyanins from black soybean seed coats protected Hacats from UVB-induced apoptosis. Lee et al. [16] reported that the processed Panax ginseng, Sun Ginseng, has an anti-apoptotic effect on UVB-irradiated Hacats. The present study indicated that AAH had much lower toxicity on Hacats than the positive control matrixyl (81.52% vs. 5.32%). Additionally, AAH possessed significantly stronger ability (*p* < 0.05) to proliferate UVB-damaged Hacats than the positive control matrixyl (137.14% vs. 121.56%). This suggests better potential of AAH as a skin care agent in terms of toxicity and protective effect on keratinocytes, compared to the positive control matrixyl.

Following keratinocytes experiment, the protective effect of AAH on mice skin was tested. The specific characteristics of photoaging included epidermal thickening and inflammatory infiltration [17]. Indeed, Feng et al. [18] reported that UV irradiation increased, by over 2-fold, the epidermal thickness of mice, while patchouli alcohol, the major active sesquiterpene found in *Pogostemonis Herba*, decreased by over 20% of the epidermal thickness. Meanwhile, UV exposure led to severe wrinkling with deep furrows, laxity and erythema in the skin of mice, but patchouli alcohol inhibited the formation of UV-induced erythema, roughness, and deep wrinkles. Similarly, Wu et al. [19] found that UVB resulted in an over 3-fold increase in the skin thickness compared with that of normal mice, and induced erythema and inflammation of the mice skin, *Coffea arabica* extract decreased the epidermal thickness by 30%, and ameliorated UVB-induced inflammation and erythema. Our data also indicated that the thickness of mice skin after UVB irradiation (40.2 µm) was dramatically increased, over 3-fold compared to the thickness of normal mice skin (13.1 µm), while AAH reversed the increased thickness of mice skin (13.5 µm). On the other hand, UVB-irradiated mice skin displayed vacuolar degeneration of epidermal basal cells and inflammatory cells infiltration of the dermis, while AAH removed the vacuolar degeneration and inflammatory cells infiltration.

It is well known that UV irradiation induces oxidative damage by inhibiting the activities of endogenous antioxidant enzymes such as SOD and GSH-Px, elevating the production of MDA (a well-known biomarker for lipid peroxidation) [20]. In addition, UV irradiation alters the connective tissues of the skin by up-regulating the expression of MMPs, which play an important role in skin photoaging. For example, by cleaving type I and type III collagen, MMP-1 initiates collagen breakdown; after being activated by MMP-3, MMP-1, -2, and -9 derived from dermal fibroblasts or keratinocytes initiate degradation of type I and III collagens [21]; MMP-2 and MMP-9 are important mediators in UV-irradiated skin damage and in the formation of wrinkles [22]. Lee et al. [23] showed that by suppressing the NF-kappaB pathway, cordycepin down-regulated MMP-1 and -3 gene expression in UVB-irradiated human dermalfibroblasts. Feng et al. [18] indicated that patchouli alcohol markedly reversed the decreased activities of SOD and GSH-Px, reduced MDA production by 30%, and inhibited the increase of MMP-1 and MMP-3 expression by about 24.6% and 39.3% respectively, in UVB-treated mice. Kim et al. [24] revealed that youngiaside increased the expression of SOD and suppressed MMP-1 production via Nrf2 and AMPK pathways in Hacats and Human Dermal Fibroblasts. Wu et al. [25] reported that *Coffea arabica* extract attenuated UVB-induced MMP-1 expression in the hairless mouse skin. The present study demonstrated that compared with the UVB-irradiated mice group, AAH reduced MDA content by 49%; increased SOD, CAT, and GSH-Px activities by 103%, 49%, and 116%, respectively; decreased MMP-1 and MMP-3 expressions by 27% and 29%, respectively; which exhibited better effects than the positive control matrixyl.

Although too many studies of UVB damage were reported, the molecular mechanisms causing this damage requires further elucidation. Over the past few years, proteomic tools have been used to investigate the biological effects of UVB exposure on human skin. For example, Bertrand-Vallery et al. [26] performed a 2D-DIGE proteomic profiling of human keratinocytes undergoing UVB-induced alternative differentiation, 69 differentially abundant proteins were identified by mass spectrometry, especially, confirming TRIpartite Motif Protein 29 as a survival factor. Wu et al. [25] employed lysine- and cysteine-labeling 2D-DIGE and MALDI-TOF mass spectrometry to conduct proteomic analysis of UVB-induced protein expression in skin fibroblasts. The results showed that 89 significantly changed proteins were identified, these UVB-modulated proteins were involved in many cellular responses including photoaging, melanogenesis, anti-apoptosis, tumorigenesis, and cell migration. Fang et al. [17] applied a high-throughput 2DE analysis coupled with MALDI-TOF MS to profile the global proteins involved in chronologically aged and photoaged skin in nude mice, and 15 differential proteins were identified. The most striking characteristic was that 14-3-3 sigma was down-regulated by 3.41-fold in the chronological aging skin, and proliferating cell nuclear antigen was up-regulated by 1.5-fold in UVB-induced aging skin.

In the present study, a novel iTRAQ-based proteomics tool was applied to obtain new insights into the protein profiles involved in photoaged mice skin. Compared with UVB treatment group, 60 differential proteins were identified in AAH treatment group. These proteins formed a complex acting network consisting of two core sub-networks and were involved in three major pathways: HIF-1 signaling pathway, glycolysis/gluconeogenesis, and methane metabolism.

Among the significantly up-regulated proteins, the proteins with a little link to aging include ODPA (Pyruvate dehydrogenase E1 component subunit alpha) (1.6 fold) and AT2A1 (Sarcoplasmic/endoplasmic reticulum calcium ATPase 1, SERCA1) (1.6 fold). It was reported that pyruvate dehydrogenase was decreased in senescent skin fibroblasts [27]; increased expression of SERCA can reduce hydroxyl radical injury in murine myocardium [28]. The most remarkedly down-regulated protein was HPT (Haptoglobin), which was 3.4-fold reduced in the modified hexapeptide group compared to the UVB group. Haptoglobin is an acute-phase protein secreted by white adipose tissue or liver cells. HPT is induced in pro-oxidative conditions such as systemic inflammation or obesity. In both inflammation and obesity HPT is up-regulated [29]. K2C6A (Keratin 6A) was also a remarkedly altered protein with 3.2-fold reduction. Similarly, other keratins were also decreased, including K1C17 (keratin 17) (−1.8 fold), K1C28 (keratin 28) (−1.8 fold), K1C27 (keratin 27) (−1.7 fold), K1C16 (keratin 16) (−1.6 fold), and K2C71 (keratin 71) (−2.1 fold). It was reported that keratin-6 was significantly higher (*p* < 0.05) in elderly skin [30]. The aging of skin is associated with decreased barrier function and gradual deterioration of the epidermal immune response, leading to chronic inflammation. Depianto et al. [31] pointed out that absence of keratin 17 attenuated hyperplasia and inflammation in models of acute dermatitis. High expression of keratin 16 in mice skin could cause skin lesions and alterations in keratin filament organization and in cell adhesion [32]. Thus, the above-mentioned disappearance of inflammatory cells infiltration could be related to attenuation of skin inflammation caused by significant down-regulation of HPT, K2C6A, and K1C17.

In conclusion, this is the first study to demonstrate the anti-photoaging effects of an acetylated and amidated peptide in UVB-irradiated keratinocytes and mice. The results showed that AAH reduced the content of MDA, increased the activities of SOD, CAT, and GSH-Px, and decreased the expression of MMP-1 and MMP-3. By employing a novel iTRAQ-based proteomic analysis, 60 differential proteins were identified, which were mapped into an interaction network composed of two core sub-networks, and key pathways were determined. In a word, the present data suggests that AAH was superior to the positive control matrixyl and has strong potential to be developed into a cosmetic product against skin photoaging.

## Figures and Tables

**Table 1:** Differential proteins related to the protection effect of the acetylated and amidated hexapeptide.

Accession	B	D	FC (D/B)	log2FC (D/B)	*p*-Value	Significant	Regulate
ANK3_MOUSE	0.91623	1.196753	1.306171	0.385343	0.000904	yes	up
CO6A1_MOUSE	0.827951	0.345235	0.416976	−1.26197	0.002326	yes	down
HSPB6_MOUSE	0.625411	1.224668	1.95818	0.969513	0.002556	yes	up
RS21_MOUSE	2.443534	0.794362	0.325087	−1.6211	0.003971	yes	down
GPDM_MOUSE	0.82043	1.132509	1.380384	0.46507	0.00559	yes	up
RS3A_MOUSE	1.185781	0.698351	0.588937	−0.76381	0.00586	yes	down
CH60_MOUSE	1.343677	0.492071	0.366212	−1.44925	0.00646	yes	down
K1C17_MOUSE	1.31876	0.385973	0.292679	−1.77261	0.006467	yes	down
HPT_MOUSE	3.841142	0.372877	0.097075	−3.36476	0.006876	yes	down
NF1_MOUSE	0.887495	1.288372	1.451696	0.537739	0.007991	yes	up
ENOB_MOUSE	0.373393	1.004882	2.69122	1.42826	0.008388	yes	up
A2M_MOUSE	1.374173	1.076648	0.783488	−0.35202	0.008462	yes	down
CAH3_MOUSE	0.402064	0.995416	2.475768	1.307876	0.008729	yes	up
K1C28_MOUSE	2.180505	0.634469	0.290974	−1.78104	0.009607	yes	down
PYGM_MOUSE	0.245837	0.912978	3.713754	1.892878	0.00997	yes	up
NPM_MOUSE	1.63725	1.019672	0.622795	-0.68317	0.010896	yes	down
LDHA_MOUSE	0.724713	1.004712	1.386359	0.471301	0.011192	yes	up
TRFE_MOUSE	1.653381	0.731186	0.442237	−1.17711	0.011198	yes	down
ODPA_MOUSE	0.34843	1.081721	3.104561	1.634389	0.012074	yes	up
MURC_MOUSE	0.539877	0.805455	1.491924	0.577174	0.016378	yes	up
TXNL1_MOUSE	1.253474	0.942139	0.751623	−0.41192	0.016452	yes	down
EPN3_MOUSE	0.822867	1.572029	1.910429	0.933897	0.016926	yes	up
ANXA2_MOUSE	0.78758	0.451204	0.572899	−0.80365	0.01695	yes	down
MDHC_MOUSE	0.737936	0.899583	1.219054	0.285762	0.017653	yes	up
SYNP2_MOUSE	0.546814	1.127627	2.062178	1.044169	0.01788	yes	up
ATPO_MOUSE	0.899583	2.080248	2.312457	1.209427	0.017984	yes	up
DEST_MOUSE	1.349091	0.843344	0.62512	−0.6778	0.018374	yes	down
VATB2_MOUSE	1.616824	0.737951	0.45642	−1.13156	0.018838	yes	down
MLRS_MOUSE	0.644024	1.264857	1.963991	0.973788	0.02064	yes	up
AMPL_MOUSE	1.507006	1.005905	0.667486	−0.58319	0.021085	yes	down
GLYG_MOUSE	0.676148	0.824278	1.21908	0.285793	0.023871	yes	up
AT2A1_MOUSE	2.670282	7.878805	2.950552	1.560985	0.025035	yes	up
K1C27_MOUSE	1.387947	0.415728	0.299527	−1.73924	0.025627	yes	down
ITIH3_MOUSE	1.978311	0.867782	0.438648	−1.18887	0.028266	yes	down
RS11_MOUSE	0.805592	0.505846	0.627919	−0.67135	0.029542	yes	down
RM11_MOUSE	0.883314	1.086471	1.229995	0.298652	0.029977	yes	up
RBMS2_MOUSE	0.739031	1.081721	1.463701	0.549621	0.030714	yes	up
CYC_MOUSE	0.594349	1.33729	2.250008	1.16993	0.031063	yes	up
K1C16_MOUSE	1.231848	0.41048	0.333223	−1.58544	0.031209	yes	down
K2C71_MOUSE	1.331865	0.313541	0.235415	−2.08672	0.032436	yes	down
K2C6A_MOUSE	3.102133	0.347336	0.111967	−3.15885	0.033321	yes	down
HSPB2_MOUSE	0.563349	1.001528	1.777809	0.8301	0.033797	yes	up
NNTM_MOUSE	0.973119	1.247436	1.281895	0.358278	0.033941	yes	up
COX41_MOUSE	1.271059	1.889273	1.486377	0.5718	0.034524	yes	up
GCAB_MOUSE	0.542972	0.317315	0.584403	−0.77496	0.035724	yes	down
ALDOA_MOUSE	0.217516	0.558516	2.567704	1.360479	0.03765	yes	up
PDLI5_MOUSE	0.507804	1.323852	2.607016	1.382399	0.038285	yes	up
PGAM2_MOUSE	0.544537	1.247436	2.290818	1.195863	0.038803	yes	up
NUCL_MOUSE	1.214212	0.917008	0.755229	−0.40501	0.038826	yes	down
TITIN_MOUSE	0.525364	1.998309	3.803666	1.927391	0.0389	yes	up
NDUB4_MOUSE	0.711696	0.887495	1.247013	0.318477	0.039585	yes	up
TM9S1_MOUSE	0.661394	0.956005	1.44544	0.531508	0.040421	yes	up
LRC15_MOUSE	0.769529	1.380911	1.794488	0.843572	0.042306	yes	up
GRP78_MOUSE	1.316223	0.519738	0.39487	−1.34055	0.044264	yes	down
MYOZ3_MOUSE	0.498299	0.948807	1.90409	0.929101	0.0481	yes	up
EVPL_MOUSE	1.009253	0.708314	0.70182	−0.51083	0.048194	yes	down
ECP3_MOUSE	0.732769	2.571728	3.509603	1.811308	0.048229	yes	up
KR151_MOUSE	0.748812	0.476557	0.636418	−0.65195	0.048479	yes	down
F16P2_MOUSE	0.676664	1.004627	1.484676	0.570148	0.04869	yes	up
K2C73_MOUSE	1.303273	0.655997	0.503346	−0.99038	0.049896	yes	down

**Table 2:** Validation of differential proteins from iTRAQ by Western blot (WB).

Protein Name	D/B	
	iTRAQ	WB
Haptoglobin	−3.36	−2.33
Cytochrome c	1.17	1.23
Nucleophosmin	−0.68	−1.22
HSP60	−1.45	−1.16
CA3	1.31	0.75
PDHA1	1.63	1.04

## References

[1] Na C.R., Wang S., Kirsner R.S., Federman D.G. (2012). Elderly adults and skin disorders: Common problems for nondermatologists. South Med. J..

[2] Kong S.Z., Shi X.G., Feng X.X., Li W.J., Liu W.H., Chen Z.W., Xie J.H., Lai X.P., Zhang S.X., Zhang X.J. (2013). Inhibitory effect of hydroxysafflor yellow a on mouse skin photoaging induced by ultraviolet irradiation. Rejuvenation Res..

[3] Brenneisen P., Sies H., Scharffetter-Kochanek K. (2002). Ultraviolet-B irradiation and matrix metalloproteinases: From induction via signaling to initial events. Ann. N. Y. Acad. Sci..

[4] Debacq-Chainiaux F., Borlon C., De Hertogh B., Remacle J., Morvan P.Y., Vallée R., Toussaint O. (2006). Identification of potential anti-photoageing algal compounds using an in-vitro model of photoageing. J. Pharm Pharmacol..

[5] Neyrinck A.M., Taminiau B., Walgrave H., Daube G., Cani P.D., Bindels L.B., Delzenne N.M. (2017). Spirulina Protects against Hepatic Inflammation in Aging: An Effect Related to the Modulation of the Gut Microbiota?. Nutrients.

[6] Sadek K.M., Lebda M.A., Nasr S.M., Shoukry M. (2017). Spirulina platensis prevents hyperglycemia in rats by modulating gluconeogenesis and apoptosis via modification of oxidative stress and MAPK-pathways. Biomed. Pharmacother..

[7] Wu Q., Liu L., Miron A., Klímová B., Wan D., Kuca K. (2016). The antioxidant, immunomodulatory, and antiinflammatory activities of Spirulina: An overview. Arch. Toxicol..

[8] Souza C., Campos P.M., Schanzer S., Albrecht S., Lohan S.B., Lademann J., Darvin M.E., Meinke M.C. (2017). Radical scavenging activity of a sunscreen enriched by antioxidants providing protection in the whole solar spectral range. Eur. J. Pharm Sci..

[9] Zeng Q.H., Fan X.D., Zheng Q.P., Wang J.J., Zhang X.W. (2018). Anti-oxidant, hemolysis inhibition, and collagen-stimulating activities of a new hexapeptide derived from *Arthrospira (Spirulina) platensis*. J. Appl. Phycol..

[10] Wallace R.J. (1992). Acetylation of peptides inhibits their degradation by rumen micro-organisms. Br. J. Nutr..

[11] Kim K.H., Seong B.L. (2001). Peptide Amidation: Production of Peptide Hormones in vivo and *in vitro*. Biotechnol. Bioprocess. Eng..

[12] Thomas A. (2011). Towards a Functional Understanding of Protein N-Terminal Acetylation. PLoS Biol..

[13] Song K.H., Kim S.B., Shim C.K., Chung S.J., Kim D.D., Rhee S.K., Choi G.J., Kim C.H., Kim K. (2015). Paracellular permeation-enhancing effect of AT1002 C-terminal amidation in nasal delivery. Drug Des. Devel Ther..

[14] Sheehan J.M. (2002). Young AR the sunburn cell revisited: An update on mechanistic aspects. Photochem. Photobiol. Sci..

[15] Tsoyi K., Park H.B., Kim Y.M., Chung J.I., Shin S.C., Shim H.J., Lee W.S., Seo H.G., Lee J.H., Chang K.C. (2008). Protective effect of anthocyanins from black soybean seed coats on UVB-induced apoptotic cell death in vitro and in vivo. J. Agric. Food Chem..

[16] Lee H., Lee J.Y., Song K.C., Kim J., Park J.H., Chun K.H., Hwang G.S. (2012). Protective Effect of Processed Panax ginseng, Sun Ginseng on UVB-irradiated Human Skin Keratinocyte and Human Dermal Fibroblast. J. Ginseng Res..

[17] Fang J.Y., Wang P.W., Huang C.H., Chen M.H., Wu Y.R., Pan T.L. (2016). Skin aging caused by intrinsic or extrinsic processes characterized with functional proteomics. Proteomics.

[18] Feng X.X., Yu X.T., Li W.J., Kong S.Z., Liu Y.H., Zhang X., Xian Y.F., Zhang X.J., Su Z.R., Lin Z.X. (2014). Effects of topical application of patchouli alcohol on the UV-induced skin photoaging in mice. Eur. J. Pharm Sci..

[19] Wu P.Y., Huang C.C., Chu Y., Huang Y.H., Lin P., Liu Y.H., Wen K.C., Lin C.Y., Hsu M.C., Chiang H.M. (2017). Alleviation of Ultraviolet B-Induced Photodamage by Coffea arabica Extract in Human Skin Fibroblasts and Hairless Mouse Skin. Int. J. Mol. Sci..

[20] Pillai S., Oresajo C., Hayward J. (2005). Ultraviolet radiation and skin aging: Roles of reactive oxygen species, inflammation and protease activation, and strategies for prevention of inflammation-induced matrix degradation—A review. Int. J. Cosmet. Sci..

[21] Nagase H., Visse R., Murphy G. (2006). Structure and function of matrix metalloproteinases and TIMPs. Cardiovasc. Res..

[22] Steinbrenner H., Ramos M.C., Stuhlmann D., Sies H., Brenneisen P. (2003). UVA-mediated downregulation of MMP-2 and MMP-9 in human epidermal keratinocytes. Biochem. Biophys. Res. Commun..

[23] Lee Y.R., Noh E.M., Jeong E.Y., Yun S.K., Jeong Y.J., Kim J.H., Kwon K.B., Kim B.S., Lee S.H., Park C.S. (2009). Cordycepin inhibits UVB-induced matrix metalloproteinase expression by suppressing the NF-kappaB pathway in human dermal fibroblasts. Exp. Mol. Med..

[24] Kim M., Park Y.G., Lee H.J., Lim S.J., Nho C.W. (2015). Youngiasides A and C Isolated from Youngia denticulatum Inhibit UVB-Induced MMP Expression and Promote Type I Procollagen Production via Repression of MAPK/AP-1/NF-κB and Activation of AMPK/Nrf2 in HaCaT Cells and Human Dermal Fibroblasts. J. Agric. Food Chem..

[25] Wu C.L., Chou H.C., Cheng C.S., Li J.M., Lin S.T., Chen Y.W., Chan H.L. (2012). Proteomic analysis of UVB-induced protein expression-and redox-dependent changes in skin fibroblasts using lysine- and cysteine-labeling two-dimensional difference gel electrophoresis. J. Proteom..

[26] Bertrand-Vallery V., Belot N., Dieu M., Delaive E., Ninane N., Demazy C., Raes M., Salmon M., Poumay Y., Debacq-Chainiaux F. (2010). Proteomic profiling of human keratinocytes undergoing UVB-induced alternative differentiation reveals TRIpartite Motif Protein 29 as a survival factor. PLoS ONE.

[27] Wei Y.H., Wu S.B., Ma Y.S., Lee H.C. (2009). Respiratory function decline and DNA mutation in mitochondria, oxidative stress and altered gene expression during aging. Chang Gung Med. J..

[28] Hiranandani N., Bupha-Intr T., Janssen P.M. (2006). SERCA overexpression reduces hydroxyl radical injury in murine myocardium. Am. J. Physiol Heart Circ. Physiol..

[29] Lisi S., Gamucci O., Vottari T., Scabia G., Funicello M., Marchi M., Galli G., Arisi I., Brandi R., D’Onofrio M. (2011). Obesity-associated hepatosteatosis and impairment of glucose homeostasis are attenuated by haptoglobin deficiency. Diabetes.

[30] Kinn P.M., Holdren G.O., Westermeyer B.A., Abuissa M., Fischer C.L., Fairley J.A., Brogden K.A., Brogden N.K. (2015). Age-dependent variation in cytokines, chemokines, and biologic analytes rinsed from the surface of healthy human skin. Sci. Rep..

[31] Depianto D., Kerns M.L., Dlugosz A.A., Coulombe P.A. (2010). Keratin 17 promotes epithelial proliferation and tumor growth by polarizing the immune response in skin. Nat. Genet..

[32] Wawersik M.J., Mazzalupo S., Nguyen D., Coulombe P.A. (2001). Increased levels of keratin 16 alter epithelialization potential of mouse skin keratinocytes in vivo and ex vivo. Mol. Biol. Cell..

